# New Models for Partnering With the Business Sector: An Interview With Gene Matthews

**Published:** 2009-03-15

**Authors:** Elizabeth Majestic

**Affiliations:** Centers for Disease Control and Prevention

Gene Matthews, former chief counsel at the Centers for Disease Control and Prevention (CDC) and a senior fellow with the North Carolina Institute for Public Health, was interviewed by Elizabeth Majestic with CDC's National Center for Chronic Disease Prevention and Health Promotion for *Preventing Chronic Disease*, CDC's online journal on public health policy, practice, and research (http://www.cdc.gov/pcd/). The Matthews discussion focuses on the importance and challenges of connecting with private-sector stakeholders and the strategies necessary to develop private networks to assist public health in the 21st century.

The interview summarizes the successes of early private-sector partnerships with the public health community. During the past 50 years, however, public health has become more internally focused and less engaged with both the business community and the political process. Matthews suggests that in an atmosphere of doing more with less, public health has to face the rigors of accountability and to think in different ways about strategic partners and how to match purposes ethically.

The interview was filmed in November 2008.

## Segment 1: The history of public health partnerships with the business sector

**Figure:**
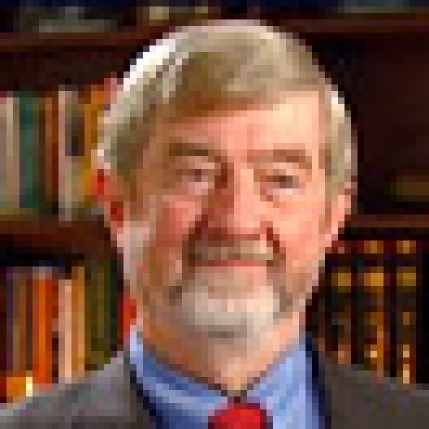


Public health has a long history of working successfully with the business sector. Initial efforts focused on providing basic health services through a partnership with the Rotary Club and expanded to fighting infectious diseases with a variety of private-sector partners. In the last 50 years, however, as public health has become increasingly isolated from the private sector, achieving public health objectives has been more difficult. Two examples of a new type of business relationship are highlighted from the National Institute of Occupational Safety and Health: 1) the tripartite agreements that called for the unions, company leaders, and CDC staff to work together to improve worker safety and 2) the National Occupational Research Agenda.

## Segment 2: Using health threats to build relationships with the business sector

**Figure:**
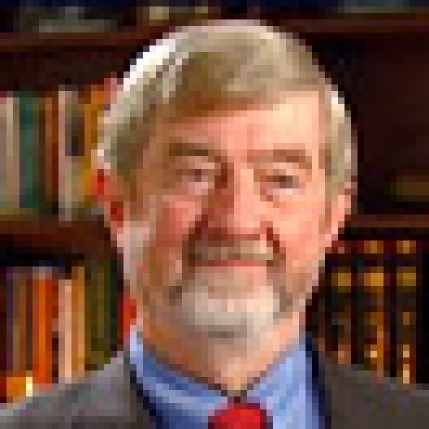


Urgent threats such as those caused by terrorists tend to unite the public health community with all sectors, including the business sector. When these threats arise, the public and private sectors provide an initial influx of resources. These resources tend to be cyclical, but they can be sustained by forming relationships that address not only the urgent health threats such as bioterrorism, but also chronic diseases.

## Segment 3: New models for partnering with the business sector

**Figure:**
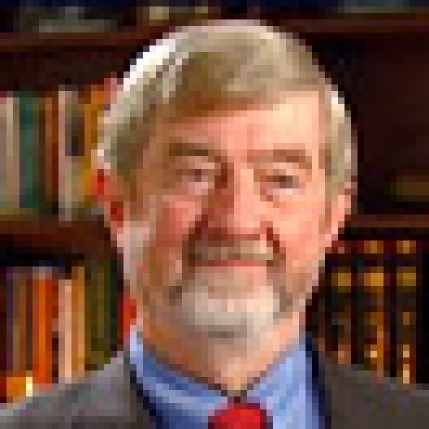


Public health needs to develop new partnership models that take into account modern health problems. By building credibility with business and community leaders on issues such as preparedness, public health can gain support for its chronic disease efforts. Initiatives that are considered desirable by communities, such as those to build recreational facilities, can help limit obesity. Public health leaders need to find this common ground.

## Segment 4: Ethics of partnerships with the private sector

**Figure:**
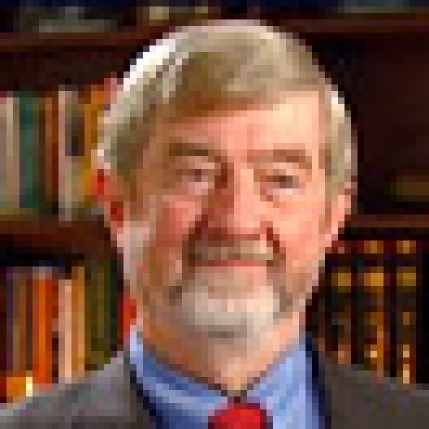


Underlying the discussion of partnerships and the debates over definitions, motives, and processes are basic questions of ethics. Which partnerships should be considered? How can their accountability be assured? To help public health professionals develop productive and ethical public-private partnerships, Matthews highlights 3 strategies that should serve as the cornerstone for partnership development.

## Segment 5: Advice for professionals working to address chronic disease

**Figure:**
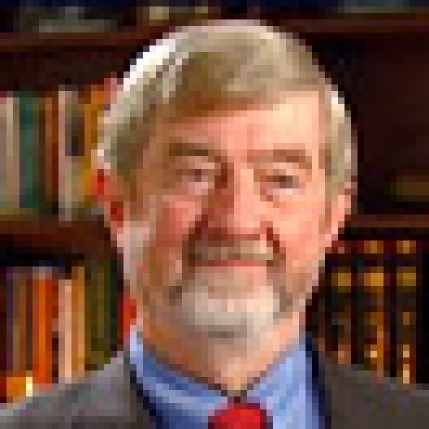


The economic downturn will increase the demands on public health. The public health community needs to prepare for the likely budget cuts, report the impact of these cuts on the public's health, and expand the range of stakeholders that influence the body politic. The Department of Agriculture is highlighted as an agency that has met these challenges by creating relationships with the private sector.

## About Mr Matthews

Gene W. Matthews is a senior fellow at the North Carolina Institute for Public Health, the outreach and service unit of the University of North Carolina School of Public Health. He also holds faculty appointments at the University of North Carolina School of Public Health and the Georgia State University College of Law. Since 1999, Mr Matthews has provided leadership in the development of CDC’s internal Public Health Law Program, an effort to reach out to the legal community and to public health practitioners. In June 2004, Mr Matthews received the Distinguished Career Award of the Public Health Law Association “… in recognition of a career devoted to using law to improve the public’s health.” Mr Matthews is a graduate of the University of North Carolina School of Law and is a member of the North Carolina Bar.

